# Dr. W. H. Deadrick

**Published:** 1858-01

**Authors:** 


Dr. William H. Deadrick.—We are called to record the death of Dr. William H. Deadrick, of Athens, Tennessee. He died on the 30th inst., at the advanced age of seventy-five years.

For half a century, Dr. Deadrick was engaged in the practice of medicine, having graduated at an early age, and entered upon the profession at Rogersville, Tennessee.

The character of his practice may be inferred from his having undertaken the novel operation, in 1810, of removing a portion of the submaxillary bone for exostosis. The boldness of this operation, as well as its success, gave Dr. Deadrick a name and a reputation as a surgeon of no mean degree, and which he had only to follow up to have placed himself at the head of the profession. But his naturally retiring disposition induced him to remain quietly in the enjoyment of his then *backwoods* practice, and to be content with the confidence of his own circle of friends and relations.

But this one operation has imprinted his name upon the records of American Surgery—especially since, with him, it was no hap-hazard case—but one directed by reason and judgment. For many years Dr. Deadrick resided at Athens, Tenn., the latter part of his life in partial blindness. He died, as he had lived, a devout Christian.—*Southern Journal of the Med. and Phys. Sciences*, Oct. 1857.

